# Circular RNAs in cancer: an emerging key player

**DOI:** 10.1186/s13045-016-0370-2

**Published:** 2017-01-03

**Authors:** Yeping Dong, Dan He, Zhenzi Peng, Wei Peng, Wenwen Shi, Jun Wang, Bin Li, Chunfang Zhang, Chaojun Duan

**Affiliations:** 1 Institute of Medical Sciences, Xiangya Hospital, Central South University, Xiangya Road 87th, Changsha, 410008 Hunan People’s Republic of China; 2 Key Laboratory of Cancer Proteomics of Chinese Ministry of Health, Xiangya Hospital, Central South University, Xiangya Road 87th, Changsha, 410008 Hunan People’s Republic of China; 3 Department of Thoracic Surgery, Xiangya Hospital, Central South University, Xiangya Road 87th, Changsha, 410008 Hunan People’s Republic of China

**Keywords:** Circular RNAs, Cancer, MicroRNA sponges, Biomarkers

## Abstract

Circular RNAs (circRNAs) are a class of endogendous RNAs that form a covalently closed continuous loop and exist extensively in mammalian cells. Majority of circRNAs are conserved across species and often show tissue/developmental stage-specific expression. CircRNAs were first thought to be the result of splicing error; however, subsequent research shows that circRNAs can function as microRNA (miRNA) sponges and regulate splicing and transcription. Emerging evidence shows that circRNAs possess closely associated with human diseases, especially cancers, and may serve as better biomarkers. After miRNA and long noncoding RNA (lncRNA), circRNAs are becoming a new hotspot in the field of RNA of cancer. Here, we review biogenesis and metabolism of circRNAs, their functions, and potential roles in cancer.

## Background

Noncoding RNAs (ncRNA) can be categorized into two subclasses, namely housekeeper ncRNAs (ribosomal RNA (rRNA), tRNA, small nuclear RNA (snRNA), snoRNA) and regulatory ncRNAs. Regulatory ncRNAs have been classified by length as follows: small noncoding RNAs (<200 bp), which includes microRNAs (miRNAs), snRNAs, piRNAs, siRNAs, and others, and long ncRNAs (lncRNAs) (>200 bp). Among the lncRNAs, circular RNAs (circRNAs) have recently emerged as a new class of endogenous RNAs that form a covalently closed continuous loop without 5′ caps and 3′ tails and exist extensively in mammalian cells. CircRNA was first found more than 20 years ago from identifying spliced transcripts of a candidate tumor suppressor gene by Nigro et al. in 1991 [1]. However, this novel type of RNA product has been thought to result from splicing errors with no function [2] and paid little attention over the next decade. Because of rapid advances in high-throughput sequencing, a large number of circRNAs have been discovered and several properties were uncovered. Firstly, circRNAs seem to be specifically expressed in tissues or developmental stage [3]. Secondly, circRNAs were more stable than associated linear mRNAs in vivo for their resistance to RNase activity [3, 4]. Thirdly, circRNAs from >14% of actively transcribed genes in human fibroblasts and in some cases the abundance of circular molecules exceeded that of associated linear mRNA by >10-fold [4]. Also, they are predominately cytoplasmic, but some seemed to be enriched in the nucleus [3–5].

### Biogenesis of circRNAs

**Fig. 1:**
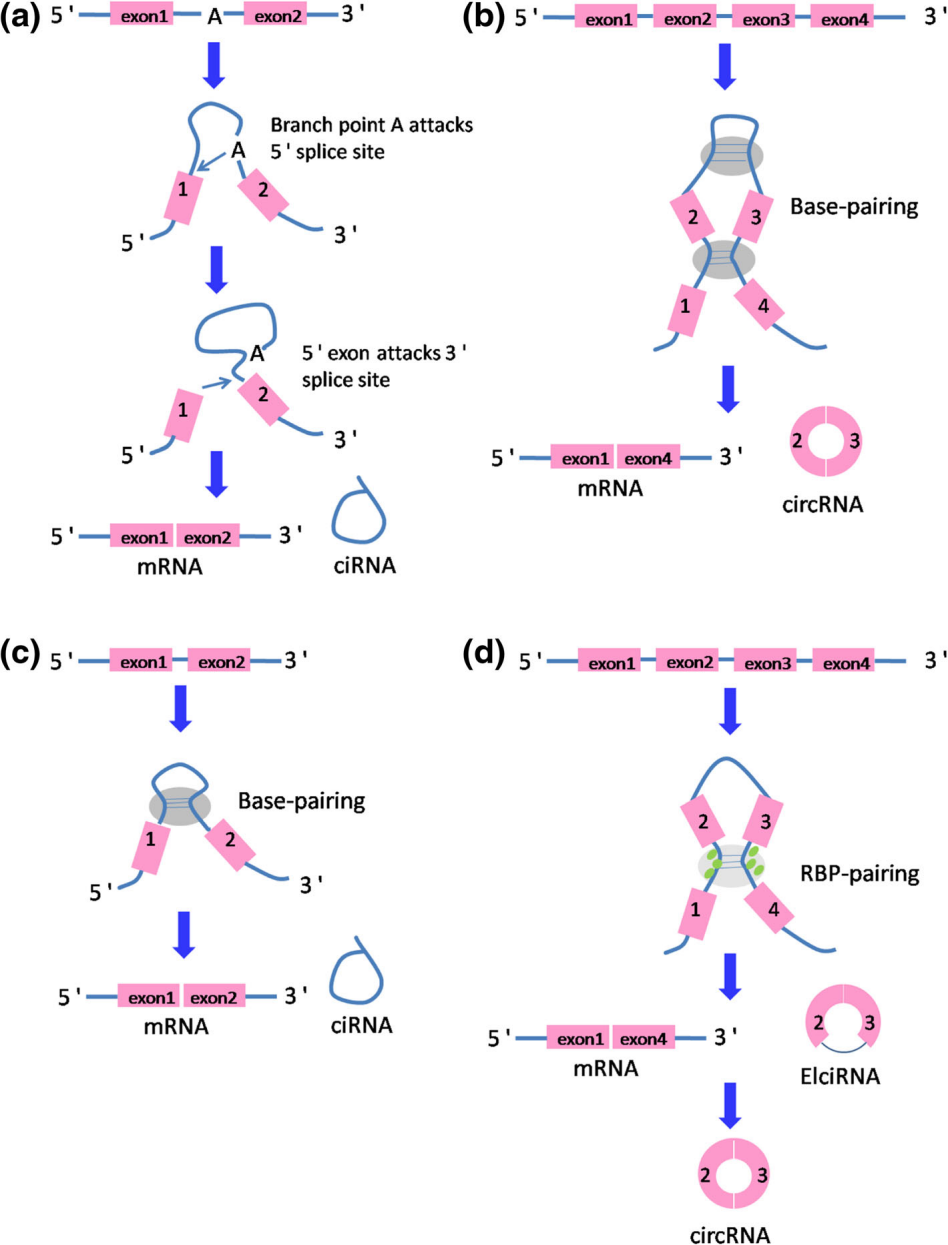
Canonical and noncanonical splicing of circRNA. **a** mRNA and ciRNA by canonical splicing. **b** circRNA by direct back splicing. **c** ciRNA by back splicing. **d** circRNA and EIciRNA by RBP-driven circularization. Abbreviations: *ciRNA* circular intronic RNA, *circRNA* exonic circular RNA, *ElciRNA* exon-intron circular RNA, *RBP* RNA-binding protein

CircRNAs have multiple origins. The majority of them are originated from exons of coding regions and the rest from 3′ UTR, 5′ UTR, introns, intergenic regions, and antisense RNAs [6]. Different from canonical splicing of linear RNAs, a single gene locus can produce various circRNAs through alternative back-splice site selection [7]. CircRNAs can be generated by both canonical and noncanonical splicing (Fig. 1). Up to now, three types of circRNAs have been identified by high-throughput sequencing: exonic circRNAs [4], circular intronic RNAs (ciRNAs) [8], and retained-intron circRNAs or ElciRNAs [9]. Exonic circRNAs account for over 80% of identified circRNAs. However, the mechanisms of biogenesis of circRNAs remain unclear. In 2013, Jeck et al. proposed two models of exonic circRNA formation [4]. One model is named “lariat-driven circularization,” and the other one is named “intronpairing-driven circularization”. It is widely accepted that back-splicing occurs in reversed orientation that connects a downstream 5′ splice site to an upstream 3′ splice site to produce circRNAs [7]. Ivanov et al. reported that circRNA formation mechanism depends on the RNA-editing enzyme adenosine deaminase acting on RNA (ADAR) [10]. Additionally, researchers reported that the RNA-binding quaking (QKI) could facilitate circRNA biogenesis during epithelial to mesenchymal transition (EMT) [11]. Recently, Gao et al. explored internal components including alternative splicing events (AS) such as exon skipping (ES), intron retention (IR), and alternative 5′ or 3′ splicing site (A5SS and A3SS) within circRNAs [12]. They demonstrated that frequency of AS events varies in different cell types, which suggest their potential roles in gene regulation.

### Biological functions of circRNAs

**Fig. 2:**
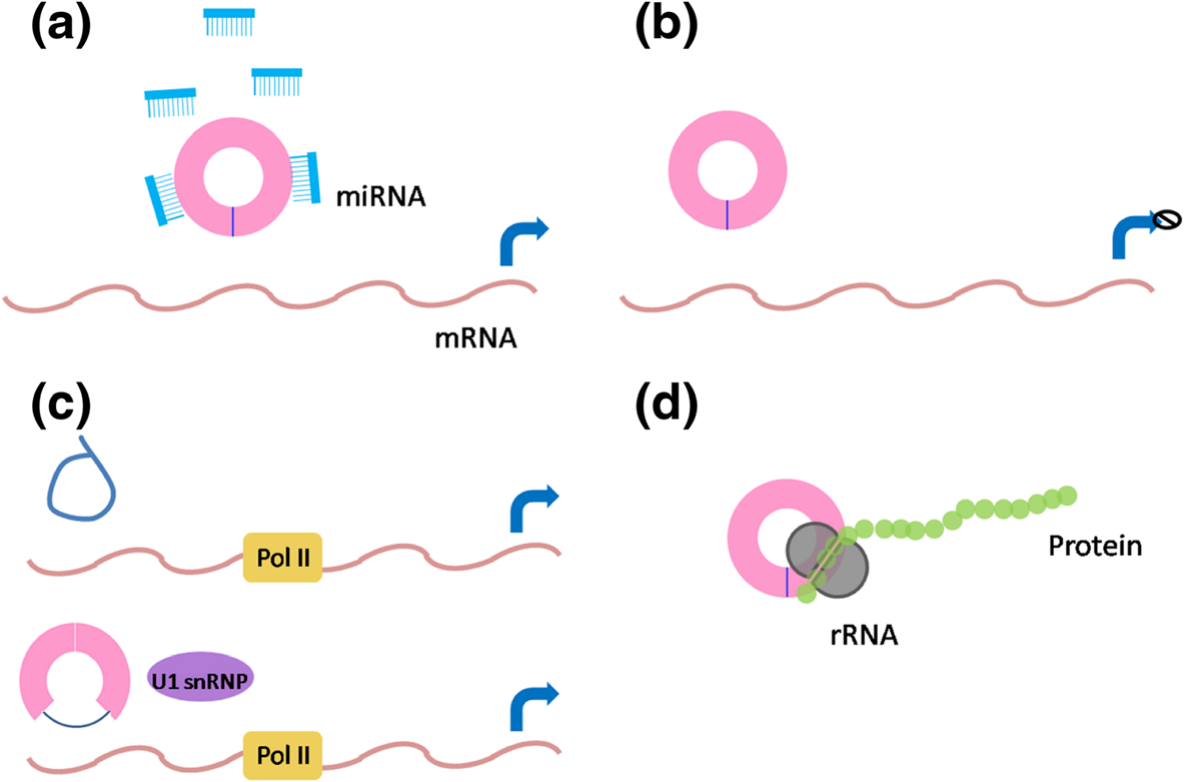
Overview of the four molecular functions of circRNA. **a** circRNAs can interact with miRNAs, acting as “sponges”. **b** circRNA have a direct role in translation. **c** ciRNA can enhance the expression of parent genes; ElciRNA can also enhance the expression of parental genes with U1 snRNP and Pol II. **d** circRNA can generate functional proteins. Abbreviations: *circRNAs* circular RNAs, *miRNA* microRNA, *ciRNA* circular intronic RNA, *Pol II* polymerase II, *ElciRNA* exon-intron circular RNA, *U1 snRNP* U1 small nuclear ribonucleoprotein particle, *mRNA* messenger RNA, *rRNA* ribosome RNA

CircRNAs can function as miRNA sponges, RNA-binding protein (RBP) sponges, and regulators of transcription, and few circRNAs can be translated into proteins/peptides (Fig. 2).

#### miRNA sponge

MiRNAs are an abundant class of short (~22 nt) noncoding RNAs that posttranscriptionally regulate gene expression via direct base pairing to target sites within mRNAs [13]. As the competitive endogenous RNAs (ceRNAs), circRNAs can compete for miRNA-binding sites. Thus, the presence of miRNAs sponges, circRNAs, can affect miRNA activities [3, 14]. For example, a circRNA named ciRS-7 or CDR1as (circular RNA sponge for miR-7 or antisense to the cerebellar degeneration-related protein 1 transcript) contains more than 70 selectively conserved miRNA target sites, and it is highly associated with Argonaute (AGO) proteins in a miR-7-dependent manner [14].

#### Regulators of transcription

Previous studies have provided evidence that circRNAs form a large number of posttranscriptional regulators [3]. Zhang and colleagues uncovered that circRNAs could regulate the expression of parental genes. They discovered that circRNAs are abundant in the nucleus with little enrichment for miRNA target sites, and knockdown of ciRNAs could lead to the reduced expression of their parental genes [3, 8]. Moreover, researchers reported after further studies that ElciRNAs, such as circEIF3J and circPAIP2, interact with U1 small nuclear ribonucleoprotein particle (U1snRNP) RNA and polymerase II (Pol II) and can enhance the transcription of their parental genes in a cis-acting manner [15]. The similar trend was also observed in circRNA PAIP2 and its parental gene [16].

From the above, we speculate that transcription activation may be a general function of circular RNAs containing intronic sequences, such as ciRNAs and EIciRNAs, and their regulatory functions may explain the abundance of ciRNAs and EIciRNAs in the nucleus.

#### Translation of circRNAs into protein/peptide

As some circRNAs carry open reading frames, one may speculate that they are translated into peptides. Early in 1998, Perriman et al. reported that a circular mRNA containing a simple green fluorescent protein (GFP) open reading frame could direct GFP expression in *Escherichia coli* [16]. Subsequently, AbouHaidar et al. discovered a circRNA (220 nt) of the virusoid associated with rice yellow mottle virus can code for a 16-kD protein [17]. It was shown that peptides can be translated from circRNAs in vitro [18] or in vivo [16], only when the RNAs contain internal ribosome entry site elements (IRES) [18] or prokaryotic ribosome-binding sites [16]. Recently, a circRNA database, named circRNADb, containing 32,914 human exonic circRNAs was established [19]. It provided detailed information of the circRNAs, including genome sequence, IRES, and open reading frame (ORF), for users to predict the translatability of certain circRNAs. However, there is no experimental evidence to prove that spliceosome-generated ecircRNAs can serve as mRNAs.

Besides serving as miRNA sponge and transcription regulator, circRNAs certainly serve as mRNA traps via sequestering the translation start site to regulate protein expression [20].

### Metabolism of circRNAs

CircRNAs are highly stable although the mechanisms by which cells degrade and/or clear circRNAs are unknown. Recently, researchers raised the possibility that cells can eliminate circRNAs via released vesicles such as exosomes and microvesicles when they reported secretion of multiple circRNAs from three different cell lines [21]. The result showed that circRNAs examined are enriched over their linear counterparts within extracellular vesicle (EV) preparations when compared to the producing cells, which suggests that expulsion from cells into extracellular space by EV release can be a mechanism by which cells reduce circRNAs.

## CircRNAs in cancer

Emerging evidence shows that circRNAs possess closely associated with human diseases, especially cancers. A report revealed that hundreds of circRNAs are more abundant in blood than corresponding linear mRNAs, which suggests that circRNAs could be used as new biomarkers in standard clinical blood samples [22]. Here, we discuss recent discoveries that implicate aberrant circRNAs in cancer (Table 1).

**Table 1:** Summary of circRNAs in cancer

Cancer name	CircRNA name	Expression level	Scope	Sample	Intersection molecules and/or pathway	References
Bladder carcinoma	circTCF25	Up	Bladder carcinoma vs normal	Tissues (*n* = 40)	miR-103a-3p,miR-107; circTCF25-miR-103a-3p/miR-107-CDK6	Zhong et al. [28]
cSCC	hsa_circ_0035381	Up	cSCC vs normal	Tissues (*n* = 12)	hsa-miR-124-5p,hsa-miR-9-5p	Sand et al. [32]
	hsa_circ_0022383	Down			hsa-miR-124-5p	
ESCC	cir-ITCH	Down	ESCC vs normal	Tissues (*n* = 684)	miR-7, miR-17, and miR-214;Wnt/β-catenin pathway	Li et al. [34]
Gastric cancer	has_circ_002059	Down	Gastric cancer vs normal	Tissues (*n* = 101)	–	Li et al. [23]
				Plasma (*n* = 36)		
CRC	hsa_circ_001988	Down	CRC vs normal	Tissues (*n* = 31)	–	Wang et al. [24]
	cir-ITCH	Down	CRC vs normal	Tissues (*n* = 45)	Wnt/β-catenin pathway	Huang et al. [25]
HCC	hsa_circ_0001649	Down	HCC vs normal	Tissues (*n* = 89)	–	Qin et al. [26]
LSCC	hsa_circ_104912	Down	LSCC vs normal	Tissues (*n* = 52)	–	Xuan et al. [27]
	hsa_circ_100855	Up				
APL	f-circRNA	–	f-circRNA-expressing vas normal	Cells	PI3K and MAPK signal transduction pathways	Guarnerio et al. [29]

Notes: *Up* upregulated, *down* downregulated

Abbreviations: *circRNAs* circular RNAs, *cSCC* cutaneous squamous cell carcinoma, *ESCC* esophageal squamous cell carcinoma, *CRC* colorectal cancer, *HCC* hepatocellular carcinoma, *LSCC* laryngeal squamous cell cancer, *APL* acute promyelocytic leukemia

### CircRNAs and gastric cancer

A report showed that hsa_circ_002059, a typical circular RNA, was found to be significantly downregulated in gastric cancer tissues compared with paired adjacent nontumor tissues. Furthermore, its levels in plasma were found significantly different between postoperative gastric cancer patients and preoperative gastric cancer patients. Importantly, that lower expression levels were significantly correlated with distal metastasis, tumor node metastasis (TNM) stage, gender, and age. In conclusion, these results suggested that circRNAs, hsa_circ_002059, may be a potential stable biomarker for the diagnosis of gastric carcinoma [23].

This study clearly demonstrated the clinical implications of hsa_circ_002059 as a biomarker, since its expression is able to distinguish normal gastric adjacent mucosa from gastric cancer tissue. Further, this circRNA may also be used as a molecular biomarker in evaluating the effectiveness of gastric resection.

### CircRNAs and colorectal cancer

Wang et al. investigated the circRNA expression in colorectal cancer (CRC), hsa_circ_001988, was selected from next-generation sequence data base for further investigation. The findings showed that the expression of hsa_circ_001988 was decreased in tumor tissues, which suggests that hsa_circ_001988 may be a novel treatment target and a potential biomarker of colorectal cancer [24].

Another report demonstrated that circRNAs were enriched in serum exosomes and could distinguish CRC from healthy controls [6]. Additionally, the abundance of tumor-derived serum exosomal circRNAs (exo-circRNAs) in serum of xenografted mice was correlated with tumor mass, which indicates that the exo-circRNAs may have an extracellular function and significant translational potential as a circulating biomarker for cancer diagnosis. Furthermore, it is also found that cir-ITCH expression was typically downregulated in CRC, and cir-ITCH could increase the level of ITCH, which is involved in the inhibition of the Wnt/β-catenin pathway. Thus, cir-ITCH may play a role in CRC by regulating the Wnt/β-catenin pathway [25]. Studies cited above illustrate that circRNAs are promising biomarkers for CRC.

### CircRNAs and hepatocellular carcinoma

Hsa_circ_0001649 expression was significantly downregulated in hepatocellular carcinoma (HCC) tissues based on an analysis of 89 paired samples of HCC and adjacent liver tissues. The findings indicate that hsa_circ_0001649 might serve as a new potential biomarker for HCC and may play a role in tumorigenesis and metastasis of HCC [26].

### CircRNAs and laryngeal cancer

Reseachers found significant upregulation (*n* = 302) or downregulation (*n*=396) of 698 circRNAs in laryngeal squamous cell cancer (LSCC) tissues via microarray analysis of four paired LSCC tissues. They further reported hsa_circRNA_100855 as the most upregulated circRNA, and their results showed that hsa_circRNA_100855 expression was significantly higher in LSCC than in the adjacent nonneoplastic tissues [27]. Overall, the data suggest that circRNAs play an important role in the tumorigenesis of LSCC and may function as novel and stable biomarkers for the diagnosis and progress of LSCC.

### CircRNAs and bladder carcinoma

CircRNA profiling and circRNA/miRNA interactions were first studied in bladder cancer, and researchers demonstrated that overexpression of circTCF25 could downregulate miR-103a-3p and miR-107, increase cyclin-dependent kinase 6 (CDK6) expression, and promote proliferation and migration in vitro and in vivo. Their work laid the foundation to investigate the functions of circRNAs in cancers. The data also suggested that circTCF25 was a new promising marker for bladder cancer [28].

### CircRNAs and acute promyelocytic leukemia

Researchers showed that well-established cancer-associated chromosomal translocations gave rise to fusion circRNAs (f-circRNA) that were produced from transcribed exons of distinct genes affected by the translocations. And then, they analyzed the presence of f-circRNA (both f-circPR and f-circM9) in the acute promyelocytic leukemia (APL)-derived leukemic cell line NB4. The data support the notion that f-circRNA, when coupled with other oncogenic stimuli, plays an active role in favoring leukemia progression in vivo [29]. Alhasan et al. found that circRNAs are enriched in human platelets 17- to 188-fold relative to nucleated tissues. As circRNAs are tolerated to degradation by exonucleases, their abundance relative to linear RNAs can be used as a marker in place of mRNA stability in the absence of transcription [30]. Previous research showed that mRNA sequencing of tumor-educated blood platelets could distinguish cancer patients from healthy population with 96% accuracy, and the location of the primary tumor was correctly identified with 71% accuracy across six different tumor types [31]. These results suggest that circRNAs and their quantity alteration in blood platelets may play a role in the diagnosis and treatment of tumor.

### Other cancers

In a recent report, a total of 322 circRNAs were differentially expressed in cutaneous squamous cell carcinoma (cSCC) and 1603 miRNA response elements (MREs) were identified in the differentially expressed circRNAs. Results showed that circRNAs are differentially expressed in cSCC and are involved in tumor formation by interfering with cSCC relevant miRNAs via miRNA sequence complementary MREs participating in epigenetic control [32]. Another study explored that the circRNA expression signatures of PDAC are dysregulated via microarray platform. The findings indicate that circRNAs can be involved in the initiation and progression of PDAC [33]. Li et al. found that cir-ITCH expression was usually low in esophageal squamous cell carcinoma (ESCC) compared to the peritumoral tissue. As sponge of miR-7, miR-17, and miR-214, cir-ITCH might increase the level of ITCH, which hyper expression promotes ubiquitination and degradation of phosphorylated Dvl2, thereby inhibiting the Wnt/β-catenin pathway [34].

CDR1as contains more than 70 selectively conserved target sites of miR-7, and emerging evidence indicates that miR-7 can directly downregulate oncogenes. Thereby, the CDR1as/miRNA axis is likely involved in cancers such as melanoma [35], breast cancer [36], gliocytoma [37], gastric cancer [38], liver cancer [39], and non-small cell lung cancer (NSCLC) [40]. It would be interesting to uncover the function of CDR1as in cancer.

In summary, findings above indicate that circRNAs are potentially involved in cancer initiation and progression. Certain circRNAs, such as cir-ITCH, play roles in more than one type of cancers. However, most research cited above lack the clear demonstration of the molecular mechanism, and further insights into their association with cancer would be warranted. Thus, clinical implications of the circRNAs as new clinical diagnostic and prognostic markers need further studies.

## Possible mechanisms of circRNAs in cancer

### ceRNA

The competitive endogenous RNAs (ceRNAs) contain shared MREs, such as mRNAs, pseudogenes, and long noncoding RNAs (lncRNAs), and can compete for miRNA binding [41]. It is known that miRNAs have been shown to be involved in nearly all aspects of cellular functions [42] and play important roles in disease initiation and progression, especially in cancers [43, 44]. In view of circRNA-binding miRNAs to regulate their targets, circRNAs may be involved in various cancers with miRNAs. For example, CDR1as is known highly expressed and has over 60 binding sites for miR-7 [45]. Emerging evidence indicates that miR-7 can directly downregulate cancerigenic factors, including epidermal growth factor receptor (EGFR) [46], P21-activated kinase-1 (Pak1) [47], insulin receptor substrate-1 (IRS-1) [48], phosphoinositide 3-kinase catalytic subunit delta (PIK3CD) [49], and mammalian target of rapamycin (mTOR) [35]. Thereby, the CDR1as/miRNA axis is likely involved in many kinds of cancers. Recently, the first study to exploit circRNA profiling and circRNA/miRNA interactions in bladder cancer was reported, and Zhong et al. determinated the regulatory role of circTCF25-miR-103a-3p/miR-107-CDK6 axes in bladder cancer [28]. Another study also found that cir-ITCH is involved in the regulation of the Wnt/β-catenin signaling pathway in vivo, as sponge of miR-7, miR-17, miR-20a, and miR-214 [27, 36].

### CircRNA-binding proteins

It has been reported that RNA-binding proteins (RBPs), such as RNA polymerase II [8], Argonaute [3], and MBL [50], can bind to circRNAs. In addition, several studies showed that circRNAs may play a role in the genesis and development of tumor via binding key proteins associated with cell proliferation, metastasis, and apoptosis. Wu et al. demonstrated that ectopic expression of the circular RNA circ-Foxo3 repressed cell cycle progression through binding to the cell cycle proteins cyclin-dependent kinase 2 (CDK2) and cyclin-dependent kinase inhibitor 1 (or p21), resulting in the formation of a ternary complex. CDK2 interacts with cyclin A and cyclin E to facilitate cell cycle entry, while p21 inhibits these interactions and arrests cell cycle progression [51]. Thus, the formation of circ-Foxo3-p21-CDK2 ternary complex can inhabit the function of CDK2 and block cell cycle progression. Because CDK2 is involved in a variety of cancers, such as breast cancer [52], NSCLC [53], and CRC [54], it is conceivable that circ-Foxo3 takes a part in cancers above by formation of circ-Foxo3-p21-CDK2 ternary complex. Circ-Foxo3 can also bind to proteins ID1, E2F1, FAK, and HIF-1α (HIF1A), retaining them in the cytoplasm and promoting cardiac senescence [55]. These findings above indicate that circRNAs may function as decoys that modify the cellular destination and/or function of bound partners.

### CircRNAs and DNA

DNA replication is the process of producing two identical replicas from one original DNA molecule. During DNA replication, circRNAs enriched in the nucleus may interact with the opposite strand of its genomic DNA through base-pairing and thus form a DNA-RNA triple helix affecting DNA replication. It is reported that pRNA interacts with the target site of the transcription factor TTF-I, forming a DNA-RNA triplex, which simultaneously recruits DNMT3b to repress rRNA expression [56]. Also, lncRNA *ANRASSF1* interacts with a DNA-forming RNA/DNA hybrid at the transcription start site, leading to reduced transcription of RAS-association domain family member 1A (*RASSF1A*) [57]. These examples highlight that the interactions between DNA and nucleus-residing circRNAs in a manner akin to linear ncRNAs.

## Conclusions

It has been decades since circRNAs were discovered. Though previously thought to be errors in RNA splicing, circRNAs now have drawn increasing attention of scientists because of recent improvement of high-throughput sequencing technologies and bioinformatics progression. In this review, we regarded natural circRNAs as an abundant, stable, diverse, and conserved class of RNA molecules. Based on the location concerning the nearest protein-coding gene, circRNAs can be classified into three subclasses: exonic circRNAs, ciRNAs, and ElciRNAs. According to their functions, circRNAs can be categorized as sponge, translation, biomarker, and regulation molecules. Their roles made them potential biomarkers of diagnosis and prognosis and therapeutical targets.

### Perspective

Recent advances about circRNAs have been focused on their biogenesis and functions as miRNA sponges. Though there has been much progress in circRNAs, the field of their functions, related mechanisms, and degradation need to be more investigated. Actually, the field of circRNAs remains largely unexplored, such as developmental stage-specific expression, turnover, localization, and degradation. Compared with miRNA and long noncoding RNAs (lncRNAs), circRNAs are promising clinical diagnostic and prognostic markers for their stable structure. Among the three subclasses of circular RNAs, one is cytoplasmatic (circRNA) and two are located in the nucleus (ciRNAs and EIciRNA) [3–5, 8], suggesting that circular RNAs may have a multitude of epigenetic roles in the cell.

CircRNAs were considered untranslatable, but recent studies prove that most circRNAs carry open reading frames, and some of them have IRES elements. With emerging evidence, more researchers are interested in this function of circRNAs. Predictably, this will become the new frontier in research of circRNAs.

Recent report shows for the first time the presence of abundant circRNAs in exosomes [6]. Exosomes are small membrane vesicles of endocytic origin secreted by most cell types [58]. The sorting of circRNAs to exosomes may be regulated by changes of associated miRNA levels in producer cells and may transfer biological activity to recipient cells. Undoubtedly, circRNAs have the potential to become clinical diagnostic and prognostic markers, and results above lay the foundation for development of circRNAs as a new class of exosome-based cancer biomarkers and suggest the potential biological function of exosomal circRNAs.

With the development of technology and research, further studies will reveal the functions of the vast majority of circRNAs in physiological and pathological processes. Furthermore, circRNAs will play a crucial role in the diagnosis and treatment of cancer.

## Abbreviations

A3SS: Alternative 3′ splicing site
A5SS: Alternative 5′ splicing site
ADAR: Enzyme adenosine deaminase acting on RNA
AGO: Argonaute
APL: Acute promyelocytic leukemia
AS: Alternative splicing events
CDK2: Cyclin-dependent kinase
CDK6: Cyclin-dependent kinase 6
CDR1as: Antisense to the cerebellar degeneration-related protein1 transcript
ceRNAs: Competing endogenous RNAs
circRNAs: Circular RNAs
ciRNA: Circular intronic RNA
ciRS-7: Circular RNA sponge for miR-7
CRC: Colorectal cancer
cSCC: Cutaneous squamous cell carcinoma
EGFR: Epidermal growth factor receptor
ElciRNA: Exon-intron circular RNA
EMT: Mesenchymal transition
ES: Exon skipping
ESCC: Esophageal squamous cell carcinoma
EVs: Extracellular vesicle
exo-circRNAs: Exosomal circRNAs
f-circRNA: Fusion circRNAs
HCC: Hepatocellular carcinoma
IR: Intron retention
IRES: Internal ribosomal entry sites
IRS-1: Insulin receptor substrate-1
lncRNA: Long noncoding RNA
LSCC: Laryngeal squamous cell cancer
miRNA: MicroRNA
MREs: MiRNA response elements
mTOR: Mammalian target of rapamycin
ncRNA: Noncoding RNAs
NSCLC: Non-small cell lung cancer
Pak1: P21-activated kinase-1
PDAC: Pancreatic ductal adenocarcinoma
PIK3CD: Phosphoinositide 3-kinase catalytic subunit delta
Pol II: RNA polymerase II
QKI: RNA-binding quaking
RASSF1A: RAS-association domain family member 1A
RBP: RNA-binding protein
rRNA: Ribosomal RNA
snRNA: Small nuclear RNA
TNM: Tumor node metastasis
U1 snRNP: U1 small nuclear ribonucleoprotein particle
